# Single-cell transcriptomics of cardiac progenitors reveals functional subpopulations and their cooperative crosstalk in cardiac repair

**DOI:** 10.1007/s13238-020-00788-6

**Published:** 2020-11-07

**Authors:** Lei Gao, Hongjie Zhang, Jingyi Cui, Lijuan Pei, Shiqi Huang, Yaning Mao, Zhongmin Liu, Ke Wei, Hongming Zhu

**Affiliations:** 1 Translational Medical Center for Stem Cell Therapy, Institute for Regenerative Medicine, Shanghai East Hospital, Tongji University School of Medicine, Shanghai, 200120 China; 2 Institute for Regenerative Medicine, Shanghai East Hospital, Shanghai Institute of Stem Cell Research and Clinical Translation, Shanghai Key Laboratory of Signaling and Disease Research, Frontier Science Center for Stem Cell Research, School of Life Sciences and Technology, Tongji University, Shanghai, 200092 China

**Dear Editor,**

Myocardial infarction is one of the leading causes of morbidity and mortality. Stem/progenitor cells therapy has emerged as a promising strategy for the cardiac repair, especially those derived from cardiac tissue, have attracted worldwide attention (Tompkins et al., 2018). However, challenges and controversies remain in characterizing functional progenitors and explaining their mechanisms of action.

Cardiosphere-derived cells (CDCs) are the only type of cardiac progenitor that has been demonstrated to be reparative for multiple heart diseases in both laboratory researches and clinical trials (Cambier et al., 2017; Sano et al., 2018). CDCs are generated based on their sphere formation capability without extra marker-based purification. Dissecting the heterogeneity of CDCs may provide novel insights into the potential relationship between their cellular composition and function, which is thereby important for clarifying current controversies over cardiac progenitors, and developing better therapies.

CDCs were isolated and characterized as reported (Figs. 1A and S1) (Cambier et al., 2017; Sano et al., 2018; Zhao et al., 2018). Using single-cell RNA sequencing (scRNA-seq), a total of 11,376 cells were profiled in an unbiased manner, and 9,621 cells were eventually analyzed after rigorous quality control (Fig. S2). As a result, 6 distinct clusters consisting of as few as 25 cells to as many as 6,054 cells per cluster were identified (Fig. 1B). Cluster-specific differentially expressed genes (DEGs) and their top GO terms were generated (Figs. 1C and S3). The potential identity of these clusters was proposed via calculating the correlation score with known cell types (Fig. S4) (Sun et al., 2019); the smallest cluster #5 was identified as endothelial-like cells, while all others are mesenchymal/stromal/fibroblast-like cells (Fig. S4). Interestingly, we found that the *Ly6a* gene (encoding stem cells antigen-1, Sca-1) was particularly expressed in some clusters, and could divide CDCs into two major subpopulations: Sca-1^+^ CDCs (cluster #0, cluster #2, and cluster #4) and Sca-1^−^ CDCs (cluster #1 and cluster #3) (Figs. 1D and S5A). Flow cytometry confirmed these two distinct groups, with the ratio of Sca-1^+^ cells in CDCs at 30% approximately (Fig. S5B and S5C).

**Figure 1:**
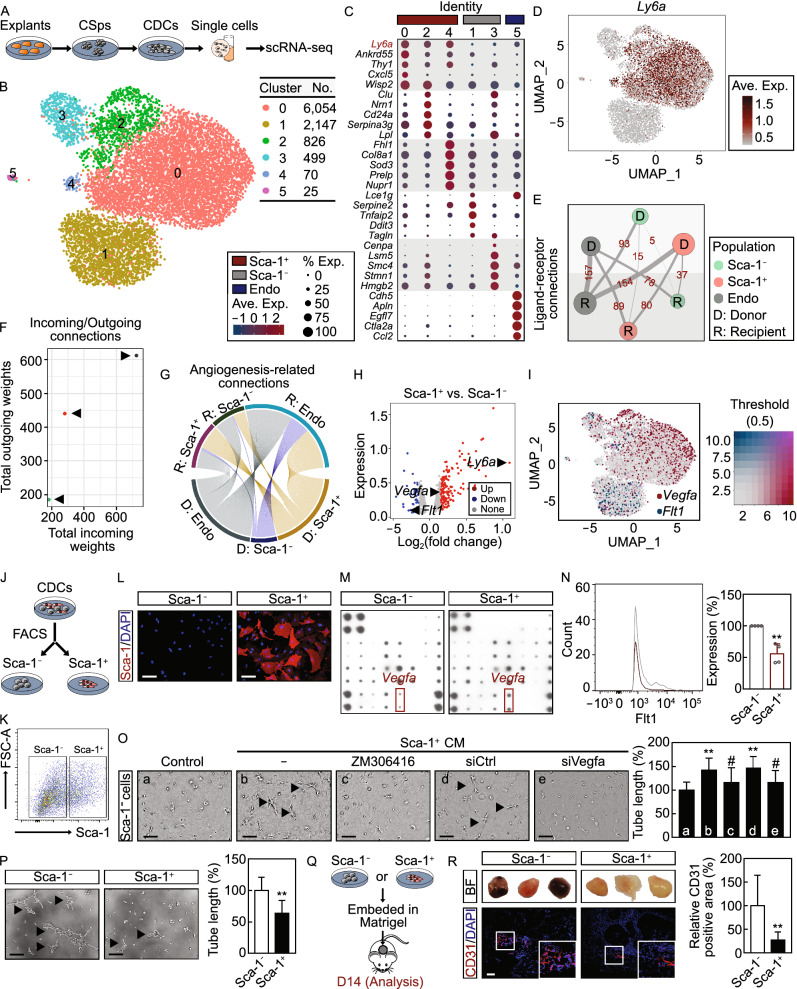
**scRNA-seq reveals two major subpopulations within CDCs**. (A) Schematic of experimental strategy. CSps, cardiospheres; CDCs, cardiosphere-derived cells; scRNA-seq, single-cell RNA sequencing. (B) Uniform manifold approximation and projection (UMAP) plot revealed that CDCs contained 6 distinct clusters with removing cell cycle genes. No., number of cells. (C) Dot plot of marker genes for each cluster. Endo, endothelial cells; Ave. Exp., average expression. (D) Feature plot showing the distribution and expression of *Ly6a* in CDCs. Network diagram of ligand-receptor connections (E); comparison of total incoming/outgoing path weights (F); network diagram of angiogenesis-related ligand-receptor connectivity among Sca-1^−^, Sca-1^+^, and endothelial cells within CDCs (G). D, donor; R, recipient. Number of interactions are annotated on the edge. (H) Volcano plot illustrating differentially expressed genes (DEGs) between Sca-1^−^ and Sca-1^+^ CDCs based on scRNA-seq dataset. Red, significantly upregulated genes; blue, significantly downregulated genes; gray, no significant difference. Fold change ≥ 2 and *P* < 0.05 were considered significant. (I) Feature plot showing the distribution and expression of *Vegfa* and *Flt1* in CDCs. Sorting strategy (J) and representative gating strategy (K) to sort Sca-1^−^ and Sca-1^+^ cells from CDCs by fluorescence-activated cell sorting (FACS). (L) Representative immunofluorescence images of sorted Sca-1^−^ and Sca-1^+^ CDCs. Cells were stained with Sca-1 (red); nuclei were labelled with DAPI (blue). Representative images of 3 independent experiments are shown. Scale bar, 100 μm. (M) Angiogenic proteome analysis of CM from Sca-1^−^ and Sca-1^+^ CDCs, respectively. CM, conditioned medium. (N) Quantification of the percentage of Flt1^+^ cells in Sca-1^−^ and Sca-1^+^ CDCs as assessed by flow cytometry. Data are shown as mean ± SD from 4 biological replicates. ^*^, significantly different from Sca-1^−^ CDCs; ^**^, *P* < 0.01. (O) Tube formation analysis of Sca-1^−^ CDCs after treatment with indicated CM. Average tube length was quantified. Data are shown as mean ± SD from 3 independent experiments. ^*^, significantly different from group a; ^**^, *P* < 0.01. ^#^, significantly different from group b; ^#^, *P* < 0.05. (P) Tube formation analysis of Sca-1^−^ and Sca-1^+^ CDCs. Data are shown as mean ± SD from 2 independent experiments. ^*^, significantly different from Sca-1^−^ CDCs; ^**^, *P* < 0.01. (Q) Experimental strategy. (R) Representative bright field (BF) and CD31 immunohistochemical images. Scale bar, 100 μm. *n* = 6 mice per group. Data are shown as mean ± SD. ^*^, significantly different from Sca-1^−^ CDCs; ^**^, *P* < 0.01

CDCs originated from mesenchymal/fibroblast cells in the heart (Fig. S4) (Cambier et al., 2017), while Sca-1 has long been considered as a stromal progenitor marker. To reveal the cellular origin of Sca-1^+^ CDC, we analyzed a published dataset of cardiac cells at various stages (Fig. S6) (DeLaughter et al., 2016), and found the proportion of Sca-1^+^ cells was increased in these mesenchymal stromal cells/fibroblast-enriched cells (MSCs/FCs) during perinatal and postnatal cardiac development (Fig. S7A–D). Specifically, a marked increase in the ratio of Sca-1^+^ CDCs-specific genes was observed in the cluster #1 of MSCs/FCs that is composed of mostly perinatal and postnatal cells, from which Sca-1^+^ CDCs may be largely originated (Fig. S7E and S7F). Collectively, our results provide novel insights into the heterogeneity of CDCs and the potential origin of Sca-1^+^ CDCs.

Cells can be classified into “donor cells” with ligands secretion and “recipient cells” with signal reception (Skelly et al., 2018). To explore if there are interactions between CDCs subpopulations, the cell-cell interaction network reflecting the strength of ligand-receptor connections was constructed; 37 connections were exported from Sca-1^+^ CDCs to Sca-1^−^ CDCs, while only 15 connections were exported from Sca-1^−^ CDCs to Sca-1^+^ CDCs (Fig. 1E). More weighted incoming and outgoing paths were also observed in Sca-1^+^ CDCs (Fig. 1F), and more angiogenesis-specific connections were also found to be sent out from Sca-1^+^ CDCs (Fig. 1G), suggesting that Sca-1^+^ CDCs is a signaling hub with more pro-angiogenic signals sent to Sca-1^−^ CDCs. As shown in Figs. 1H and S8, *Vegfa* (encoding Vegfa, an angiogenesis-related ligand), and *Flt1* (encoding Flt1, a specific receptor of Vegfa) were significantly upregulated in Sca-1^+^ and Sca-1^−^ CDCs, respectively. These results were highlighted by mapping *Vegfa* and *Flt1* across clusters identified by scRNA-seq (Fig. 1I). Sca-1^+^ and Sca-1^−^ CDCs were then sorted using Sca-1-specific antibody, and validated by immunostaining (Fig. 1J–L). Angiogenic protein array confirmed an enhanced secretion of Vegfa of Sca-1^+^ CDCs (Figs. 1M and S9) as well as other inflammation regulatory proteins such as IL-1, while flow cytometry results confirmed an elevated expression of Flt1 in Sca-1^−^ CDCs (Fig. 1N). To validate the intercellular interaction, we employed Flt1 competitive antagonist ZM306416 and *Vegfa* siRNA in the tube formation assay of Sca-1^−^ CDCs cultured in Sca-1^+^ CDCs conditioned media. As shown in Fig. 1O, both *Vegfa* knockdown in Sca-1^+^ CDCs (Fig. S10) and *Flt1* blocking in Sca-1^−^ CDCs significantly eliminated Sca-1^+^ CDC condition medium-promoted tube formation, suggesting the pro-angiogenic function of Sca-1^+^ CDCs may through an interaction between their secreted Vegfa and Flt1 receptor on Sca-1^−^ CDCs. Intriguingly, when their angiogenic capacities were directly examined, Sca-1^+^ CDCs showed less vessel-like structures *in vitro* (Fig. 1P), as well as less blood vessel formation in Matrigel plug assay *in vivo* as quantified by CD31 staining (Fig. 1Q and 1R). Collectively, these results suggest that Sca-1^+^ CDCs are more pro-angiogenic while less angiogenic itself.

In addition, there was no difference in the expression of *Klf4* and *Foxa2*, two angiogenic progenitor markers, between two subpopulations in both scRNA-seq and RNA-seq datasets (Fig. S11A and S11B). After an induction with EGM-2 medium for 14 days (Fig. S11C), both subpopulations expressed similar level of CD31 (Fig. S11D and S11E) and eNOS (Fig. S11F), while the production of NO was slightly reduced in Sca-1^+^ CDCs (Fig. S11G). In evaluation of the proliferative phenotype of subpopulations of CDCs, 8 distinct clusters were identified with cell cycle genes retained (Fig. S12A), and Sca-1 expression remained a major determinant of cluster identities with four clusters are Sca-1^+^ and three clusters are Sca-1^−^ besides endothelial-like cells (Fig. S12B). The cell cycle phases-specific score (Fig. S13A) and the proportion of cell cycle stage-specific cells (Fig. S13B) were calculated based on scRNA-seq dataset. The ratio of S phase-specific cells was lower in Sca-1^+^ CDCs (Fig. S13B), indicating that they might be less proliferative. Indeed, when compared with Sca-1^−^ CDCs, the ratio of PCNA positive cells, the number of cardiospheres formed, the proliferation rate quantified by CCK-8 assay, and the colony formation ability were significantly decreased in Sca-1^+^ CDCs (Fig. S13C–F). Consistently, RNA-seq result revealed that more cell cycle inhibitory genes were highly expressed in Sca-1^+^ CDCs (Fig. S13G). Collectively, Sca-1^+^ CDCs possess a pro-angiogenic secretion function, while Sca-1^−^ CDCs exhibit stronger angiogenic and proliferative capabilities. These results thereby prompt us to further determine which cell type harbors the cardioprotective benefit after myocardial infarction.

To determine the therapeutic effect of Sca-1^−^ and Sca-1^+^ CDCs on myocardial infarction, they were injected into mouse heart at a density of 10 × 10^4^ cells per heart immediately after coronary ligation. Only Sca-1^+^ CDCs transplantation significantly improved cardiac function (Fig. 2A), with a decreased myocardial fibrosis (Fig. 2B) and an increased angiogenesis (Fig. 2C), whereas Sca-1^−^ CDCs showed no beneficial effects. We then sought to clarify potential mechanisms of the difference in the beneficial effect between these cell types. Transcriptome analysis of isolated Sca-1^−^ and Sca-1^+^ CDCs was performed, and DEGs between Sca-1^−^ and Sca-1^+^ CDCs were obtained using 2-fold change and *P* < 0.05 as the threshold cutoff (Fig. 2D). As shown in Fig. 2E, GO analysis revealed that Sca-1^+^ CDCs-specific DEGs were highly enriched in biological processes such as angiogenesis. We also found that the majority of upregulated ligands in Sca-1^+^ CDCs were cardioprotective molecules (Fig. 2F), such as *Cxcl12* (Das et al., 2019) and *Hgf* (Ellison et al., 2011). A recent study has highlighted the important role of Wnt signaling in CDCs therapy, especially that Gata4 and β-catenin enriched in CDCs have enhanced beneficial effects (Ibrahim et al., 2019). We observed significantly upregulated expression of *Gata4* and downregulated expression of *Ctnnb1*, which encodes β-catenin in Sca-1^+^ CDCs, and one of genes that are downregulated in CDCs with high Wnt signaling, *Apcdd1*, was also lower in Sca-1^+^ CDCs, while others were not changed (Fig. S14). Thus, Wnt signaling does not seem to be a determinant of the functional differences of the Sca-1^+^ CDCs and Sca-1^−^ CDCs. It has also been well-established that extracellular vesicles (EVs) play functions in CDCs therapy (Menasché, 2018). Therefore, we also evaluated the EVs secretion of CDCs subpopulations. Through analyzing both scRNA-seq and bulk RNA-seq datasets, we found that the EVs markers were significantly enriched in Sca-1^−^ CDCs (Fig. S15), suggesting that Sca-1^−^ CDCs may be more active in EVs secretion, thus the effectiveness of Sca-1^+^ CDCs might not likely to be related to its EVs secretion. Collectively, our results suggest that the beneficial effect of Sca-1^+^ CDCs on the impaired cardiac function is largely due to their secretion of cardioprotective ligands.

**Figure 2:**
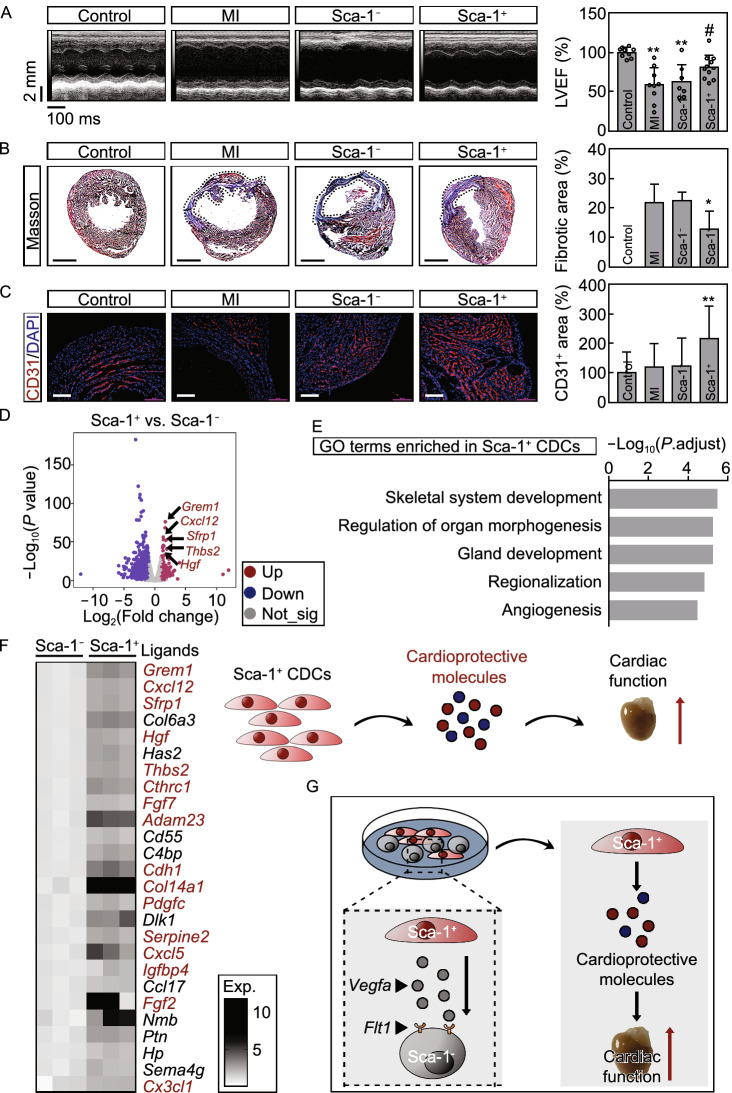
**Sca-1**^**+**^ **CDCs have beneficial effects on the cardiac function after MI**. (A) Representative images of echocardiography and echocardiographic measurements of EF. MI, myocardial infarction; LVEF, left ventricle ejection fraction. *n* = 7 mice per group at least. Data are shown as mean ± SD from 2 independent experiments. ^*^, significantly different from control; ^**^, *P* < 0.01. ^#^, significantly different from MI; ^#^, *P* < 0.05. (B) Representative images of Masson’s trichrome-stained heart sections. Scale bar, 1 mm. *n* = 4 mice per group. (C) Representative images of CD31 (red) expression in heart sections. Nuclei were labeled with DAPI (blue). Scale bar, 200 μm. *n* = 5 mice per group. Data are shown as mean ± SD. ^*^, significantly different from MI; ^*^, *P* < 0.05; ^**^, *P* < 0.01. ^#^, significantly different from Sca-1^−^ CDCs; ^#^, *P* < 0.05. (D) Volcano plot illustrating DEGs between Sca-1^−^ and Sca-1^+^ CDCs based on RNA-seq dataset. Red, significantly upregulated genes; blue, significantly downregulated genes; gray, no significant difference. Fold change ≥ 2 and *P* < 0.05 were considered significant. (E) Top 5 GO terms of significantly upregulated DEGs in Sca-1^+^ CDCs. (F) Heatmap illustrating the expression of ligands within these significantly upregulated DEGs in Sca-1^+^ CDCs. Red, reported cardioprotective molecules. (G) Schematic diagram. There is a crosstalk between Sca-1^+^ and Sca-1^−^ cells within CDCs mediated by Vegfa and Flt1. Sca-1^+^ CDCs transplantation improves the cardiac function after MI via secreting plenty of cardioprotective molecules

In conclusion, we demonstrate the heterogeneous nature of CDCs using scRNA-seq. Two major functional subsets distinguished by Sca-1 expression are identified, their potential origin and a cellular interaction between Sca-1^+^ and Sca-1^−^ CDCs mediated by Vegfa-Flt1 are also revealed. Sca-1^+^ CDCs but not Sca-1^−^ CDCs show a cardiac reparative function after myocardial infarction possibly due to their secretion of cardioprotective factors (Fig. 2G). The secretome of cardiac progenitors have been reported to hold cardioprotective effects on heart failure (Konemann et al., 2019), and further functional screen may identify the core beneficial factors in Sca-1^+^ CDCs’ secretome. Interestingly, despite having strong proliferative and angiogenic capacity *in vitro*, the Sca-1^−^ CDCs failed to repair the infarcted heart *in vivo*. Whether Sca-1^−^ CDCs require signals such as Vegfa from Sca-1^+^ CDCs for activation needs further investigation. In addition, we examined inflammation modulation (Fig. S9) as well as Wnt signaling (Fig. S14) and EVs secretion (Fig. S15) in Sca-1^+^ and Sca-1^−^ CDCs, which were suggested in previous studies (Menasché, 2018), and their differences in CDCs subpopulations emphasizes the complexity of the function of CDCs. Future research may delineate the involvement of immune-regulation, Wnt signaling, and EVs in different CDCs subpopulations.

## Electronic supplementary material

Below is the link to the electronic supplementary material.

Supplementary material 1 (PDF 4060 kb)
