# Supplementary material for: Single-cell transcriptomics of cardiac progenitors reveals functional subpopulations and their cooperative crosstalk in cardiac repair
Source: Protein Cell. 2020 Nov 7;12(2):152–7. doi: 10.1007/s13238-020-00788-6 (PMC7862461; doi:10.1007/s13238-020-00788-6)
Supplement: Supplementary file 1 — Supplementary material 1 (PDF 4060 kb) [file 13238_2020_788_MOESM1_ESM.pdf]

Supplementary Information for

Single-cell transcriptomics of cardiac progenitors reveals functional subpopulations and their cooperative crosstalk in cardiac repair

Running title: Single-cell transcriptomics of CDCs

Lei Gao[1]*, Hongjie Zhang[2]*, Jingyi Cui[1], Lijuan Pei[2], Shiqi Huang[2], Yaning Mao[1], Zhongmin Liu[1]✉, Ke Wei[2]✉, Hongming Zhu[1]✉

[1] Translational Medical Center for Stem Cell Therapy & Institute for Regenerative Medicine, Shanghai East Hospital, Tongji University School of Medicine, Shanghai 200120, P.R. China

[2] Institute for Regenerative Medicine, Shanghai East Hospital, Shanghai Key Laboratory of Signaling and Disease Research, Frontier Science Center for Stem Cell Research, School of Life Science and Technology, Tongji University, Shanghai 200092, P.R. China

✉ Correspondence: zhm@tongji.edu.cn (H. Zhu), kewei@tongji.edu.cn (K. Wei), liu.zhongmin@tongji.edu.cn (Z. Liu)

* Lei Gao and Hongjie Zhang contributed equally to this study.

Keywords: Cardiosphere-derived cells, single-cell RNA sequencing, Sca-1, myocardial infarction

Materials and methods

Animal models

Animal experiments were conducted in accordance with the Guide for the Care and Use of Laboratory Animals published by the National Institutes of Health of the United States (Eighth Edition, 2011), and approved by the Animal Care and Use Committee of Tongji University School of Medicine. C57BL/6 mice (wild-type, male, 8-10 weeks of age) were obtained from Shanghai Jiesijie Animal Company (Shanghai, China). To establish MI models, mice were anesthetized by isoflurane inhalation (1.5%); the left anterior descending branch of the coronary artery was ligated as introduced previously (Dutta et al., 2012). Mice cardiac functions were measured using the echocardiography. All measurements were performed by an assigned technician in a blind manner. Mice were euthanized by CO_2 inhalation.

Cell culture

CDCs were generated as previously reported (Cambier et al., 2017; Sano et al., 2018; Zhao et al., 2018). In brief, mouse heart was minced into small pieces and then digested enzymatically. Tissue fragments were plated as "explants" onto dishes coated with fibronectin (BD Bioscience, USA). After 7-14 days, these explants spontaneously yielded phase-bright round outgrowth cells, which were harvested using 0.05% trypsin and cultured in ultra-low attachment dishes (Corning, USA) to form cardiospheres. CDCs were obtained after seeding collected cardiospheres onto fibronectin-coated dishes. Sca-1^- CDCs and Sca-1^+ CDCs were sorted from CDCs by using the FITC-

conjugated Sca-1 antibody (Cat#ab25031, Abcam, USA). CDCs, Sca-1$^-$ CDCs, and Sca-1$^+$ CDCs were maintained in IMDM (Gibco, USA) supplemented with 20% FBS (HyClone, UK), 100 U/mL penicillin, 100 μg/mL streptomycin, and 0.1 mmol/L 2-mercaptoethanol (Gibco, USA) (Cambier et al., 2017). HUVEC was purchased from Shanghai Institute of Biochemistry and Cell Biology. HUVEC were cultured in DMEM (Gibco, USA) supplemented with 10% FBS, 100 U/mL penicillin, and 100 μg/mL streptomycin. Cells were incubated at 37 °C in a humidified atmosphere supplemented with 5% CO_2.

Single-cell RNA sequencing

Cell suspension was loaded into Chromium microfluidic chips with 3' (version 2.0) chemistry and barcoded with a 10X Chromium Controller (10X Genomics). RNA from the barcoded cells was subsequently reverse-transcribed and sequencing libraries constructed with reagents from a Chromium Single Cell 3' v2 (version 2.0) reagent kit (10X Genomics) according to the manufacturer's instructions. Sequencing was performed with Illumina (HiSeq 2000) according to the manufacturer's instructions (Illumina).

Data pre-processing

Raw sequencing data were processed by Cell Ranger (version 2.2.0, 10X Genomics) using default mapping arguments. Reads were aligned to the mm10 genome and counted with GRCm38.92 for annotation. A gene-barcode matrix was generated with

11376 cells and 28692 genes after filtering. Subsequent filtering and clustering analyses were conducted using the Seurat R package (version 3.1.0, http://satijalab.org/seurat/).

Data quality control

Several criteria were employed to filter poor-quality cells: (1) number of genes fewer than 5500 and greater than 2500; (2) number of counts detected greater than 10000 and fewer than 40000; (3) reads mapping to mitochondria fewer than 4%. Genes were filtered with fewer than 3 cells expressed. After being filtered, 9621 cells finally kept for downstream analyses.

Data normalization and scales

Data were normalized by the “LogNormalize” method with a scale factor of 10,000 using the “NormalizeData” function in Seurat. Mitochondrion genes were also scaled using the “ScaleData” function.

Clustering

2500 highly variable genes were calculated using the “FindVariableFeatures” function. Follow the dimensionality reduction by principal component analysis (PCA), top 25 principal components (PCs) were adopted as best performance for downstream analyses; uniform manifold approximation and projection (UMAP) dimensionality reduction was used for visualization in two dimensions.

Elimination of cell cycle effect

Each individual cell was assigned a cell cycle score and stage based on the expression of cell cycle phases-specific genes by the “CellCycleScoring” function. Cell cycle effect was eliminated by regressing out the cell cycle score with the “ScaleData” function.

Generation of marker genes

To obtain DEGs, the “FindMarkers” function was adopted in a default parameter. To further identify marker genes of each group, clusters were analyzed using the “FindAllMarkers” function with the Wilcoxon rank-sum test.

Construction of ligand-receptor network

To construct ligand-receptor interactions between these cell types, a previously reported source code, which was modified necessarily to match the Seurat R package (version 3.1.0), was adopted (Farbehi et al., 2019). The weight of ligand-receptor interactions was also calculated. The interaction diagram was drawn using the igraph R package (https://igraph.org/r/); the diagram of the ligand-receptor interactions of angiogenesis is drawn using the circlize R package (Gu et al., 2014).

Gene ontology analysis

Gene ontology was analyzed using the clusterProfiler R package with significance threshold set at p value < 0.05 (Yu et al., 2012).

Cell type identification of CDCs

Single-cell Mouse Cell Atlas was adopted as a reference to define the cellular types of CDCs subpopulations based on single-cell digital expression between all mouse cell types and CDCs (Sun et al., 2019). 100 cells of each subset in CDCs was randomly chosen to calculate gene average expression as integer of the values for testing. Top 3 of predicted cell types for each CDCs subpopulations was shown in heatmap plot based on their Pearson correlation coefficients.

Bulk RNA-sequencing

Bulk RNA-sequencing of Sca-1^- and Sca-1^+ CDCs was performed at Novogene (Beijing, China) using the Illumina HiSeq 2500 instrument. Reads were aligned to the mm10 reference genome using the HISAT2 software with default paired-end settings (Kim et al., 2015). After being sorted by the SAMtools (Li et al., 2009), transcripts were then assembled using the StringTie (Pertea et al., 2015). Through employing the edgeR R package (Robinson et al., 2010), genes with threshold (fold change > 2 and p value < 0.05) were considered to be differentially expressed.

Cell proliferation assays

For CCK-8 assay, cells were seeded into 96-well plate. After 0, 1, and 7 days, 10 μL CCK-8 solution (Bimake, China) was added into each well. After a 2 h incubation, the absorbance was determined at 450 nm using a microplate reader (Dynatech, USA). For colony formation assay, cells were seeded into 6-well plate at a density of 500 cells per

well. After 2 weeks, cells were fixed with 4% paraformaldehyde (PFA) and stained with crystal violet (Beyotime, China). Images were captured using a microscope (DMI3000 B, Leica). For sphere formation assay, cells were seeded into ultra-low attachment 6-well plate at a density of 5×10^4 cells per well. After 3 days, images were captured using a microscope.

Tube formation assay

Cells were seeded into 96-well plate coated with Matrigel (BD Bioscience, USA). After 5 h, images were captured using a microscope. The average tube length was quantified using the AngioTool software.

Endothelial differentiation assay

In order to compare the endothelial differentiation potential of Sca-1^- and Sca-1^+ CDCs, EGM-2 medium (Lonza, USA) was applied to induce their endothelial differentiation (Gao et al., 2020). Medium was exchanged every 3 days, and cells were collected for further analyses after 14 days of treatment. For nitric oxide (NO) production assay, cells were collected and stained with 5 μM DAF-FM DA (Beyotime, China) at 37 °C for 20 min. Afterwards, cells were washed and detected by a flow cytometry (BD biosciences, USA).

Angiogenic protein analysis

Secretion of angiogenic proteins from Sca-1^- and Sca-1^+ CDCs was detected using a Mouse Angiogenesis Array C1 (RayBiotech, USA) according to the manufacturer's instructions. Images were captured using the FlourchemQ system, and signal density was then calculated with the AlphaView software (ProteinSimple, USA).

Cell transfection

A small interfering RNA targeting Vegfa (siVegfa) and non-targeting siRNA scramble control (siCtrl) were synthesized by GenePharma (Shanghai, China), and transfected into Sca-1^+ CDCs using Lipofectamine 6000 (Beyotime, China) according to the manufacturer's instructions.

In vivo matrigel plug assay

To evaluate the angiogenesis mediated by Sca-1^- and Sca-1^+ CDCs *in vivo*, cells (1×10^6 per mouse) were resuspended in 200 μL of ice-cold Matrigel, and then implanted subcutaneously on the back of mice. Matrigel plugs were removed for analysis 14 days later.

Histology and immunofluorescence

Matrigel plugs and hearts were collected, fixed with 4% PFA, embedded in paraffin, and sectioned. For immunohistochemical analysis, Matrigel plug sections were stained with primary antibody against CD31 (Cat#ab28364, Abcam, USA). To identify fibrosis areas, the Masson's Trichrome Stain Kit (Solarbio, China) was used according to the

manufacturer’s instructions. For immunofluorescence analysis, cells were stained with anti-Sca-1 (Cat#AF1226, R&D Systems, USA) antibody and DAPI (Beyotime, China).

RNA isolation and quantitative real-time PCR

Total RNA was isolated using the TRIzol (Invitrogen, USA). cDNA libraries were synthesized by using the Reverse Transcription Reagent kit (Takara, Japan) and random primers. Samples were submitted for Real-Time PCR with SYBR Green mix (Takara, Japan), and results were normalized to *Gapdh* expression. Primers for *Gapdh* and *Cd31* were obtained from Sangon Biological Engineering (Shanghai, China), and sequences are presented in Table S1. Data were analyzed by the comparative Ct ($^{\Delta\Delta}$Ct) method to quantify relative gene expression.

Western blotting

Cells were lysed in a lysis buffer (Cell Signaling Technology, USA) supplemented with protease inhibitors (Calbiochem, USA) at 4 °C for 30 min. Total protein concentration was quantified using the BCA protein assay kit (Pierce, USA). Western blotting was performed as previously described (Zhu et al., 2014); bands were captured using the LI-COR (Odyssey, USA), and quantified using the Gel-Pro (Media Cybernetics, USA). Primary antibodies against β-actin (Cat#ab8227) and CD31 (Cat#ab28364) was purchased from Abcam (Cambridge, UK).

Statistical analysis

Results are expressed as mean ± standard deviation (SD). Multiple groups were compared by One-way ANOVA and Tukey's multiple comparison test; pair groups were compared by two-tailed Student's t-test. P values < 0.05 were considered significant. Statistical analyses were conducted using GraphPad Prism 7 (GraphPad Software Inc., USA).

REFERENCES

Cambier, L., de Couto, G., Ibrahim, A., Echavez, A.K., Valle, J., Liu, W., Kreke, M., Smith, R.R., Marban, L., and Marban, E. (2017). Y RNA fragment in extracellular vesicles confers cardioprotection via modulation of IL-10 expression and secretion. EMBO molecular medicine 9, 337-352.

Dutta, P., Courties, G., Wei, Y., Leuschner, F., Gorbatov, R., Robbins, C.S., Iwamoto, Y., Thompson, B., Carlson, A.L., Heidt, T., *et al.* (2012). Myocardial infarction accelerates atherosclerosis. Nature 487, 325-329.

Farbehi, N., Patrick, R., Dorison, A., Xaymardan, M., Janbandhu, V., Wystub-Lis, K., Ho, J.W., Nordon, R.E., and Harvey, R.P. (2019). Single-cell expression profiling reveals dynamic flux of cardiac stromal, vascular and immune cells in health and injury. eLife 8.

Gao, L., Mei, S., Zhang, S., Qin, Q., Li, H., Liao, Y., Fan, H., Liu, Z., and Zhu, H. (2020). Cardio-renal Exosomes in Myocardial Infarction Serum Regulate Proangiogenic Paracrine Signaling in Adipose Mesenchymal Stem Cells. Theranostics 10, 1060-1073.

Gu, Z., Gu, L., Eils, R., Schlesner, M., and Brors, B. (2014). circlize Implements and enhances circular visualization in R. Bioinformatics (Oxford, England) 30, 2811-2812.

Ibrahim, A.G.E., Li, C., Rogers, R., Fournier, M., Li, L., Vaturi, S.D., Antes, T., Sanchez, L., Akhmerov, A., Moseley, J.J., *et al.* (2019). Augmenting canonical Wnt

signalling in therapeutically inert cells converts them into therapeutically potent exosome factories. Nature biomedical engineering 3, 695-705.

Kalluri, R., and LeBleu, V.S. (2020). The biology, function, and biomedical applications of exosomes. Science (New York, NY) 367.

Kim, D., Langmead, B., and Salzberg, S.L. (2015). HISAT: a fast spliced aligner with low memory requirements. Nature methods 12, 357-360.

Li, H., Handsaker, B., Wysoker, A., Fennell, T., Ruan, J., Homer, N., Marth, G., Abecasis, G., and Durbin, R. (2009). The Sequence Alignment/Map format and SAMtools. Bioinformatics (Oxford, England) 25, 2078-2079.

Pertea, M., Pertea, G.M., Antonescu, C.M., Chang, T.C., Mendell, J.T., and Salzberg, S.L. (2015). StringTie enables improved reconstruction of a transcriptome from RNA-seq reads. Nature biotechnology 33, 290-295.

Robinson, M.D., McCarthy, D.J., and Smyth, G.K. (2010). edgeR: a Bioconductor package for differential expression analysis of digital gene expression data. Bioinformatics (Oxford, England) 26, 139-140.

Sano, T., Ousaka, D., Goto, T., Ishigami, S., Hirai, K., Kasahara, S., Ohtsuki, S., Sano, S., and Oh, H. (2018). Impact of Cardiac Progenitor Cells on Heart Failure and Survival in Single Ventricle Congenital Heart Disease. Circulation research 122, 994-1005.

Sun, H., Zhou, Y., Fei, L., Chen, H., and Guo, G. (2019). scMCA: A Tool to Define Mouse Cell Types Based on Single-Cell Digital Expression. Methods in molecular biology (Clifton, NJ) 1935, 91-96.

Yu, G., Wang, L.G., Han, Y., and He, Q.Y. (2012). clusterProfiler: an R package for comparing biological themes among gene clusters. Omics : a journal of integrative biology 16, 284-287.

Zhao, Z.A., Han, X., Lei, W., Li, J., Yang, Z., Wu, J., Yao, M., Lu, X.A., He, L., Chen, Y.*, et al.* (2018). Lack of Cardiac Improvement After Cardiosphere-Derived Cell Transplantation in Aging Mouse Hearts. Circulation research 123, e21-e31.

Zhu, H., Sun, A., Zou, Y., and Ge, J. (2014). Inducible metabolic adaptation promotes mesenchymal stem cell therapy for ischemia: a hypoxia-induced and glycogen-based

energy prestorage strategy. Arteriosclerosis, thrombosis, and vascular biology 34, 870-876.

Figure S1

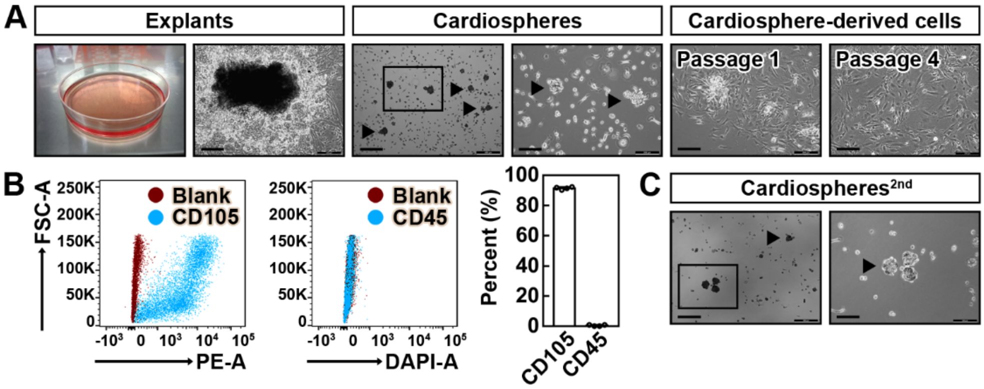

Figure S1. Generation and characterization of CDCs. (A) Mouse heart was minced into small fragments and plated as “explants” into fibronectin-coated dishes to yield outgrowth cells; outgrowth cells were then harvested and plated into ultra-low attachment dishes to self-assemble into cardiospheres; cardiospheres were collected and re-plated into fibronectin-coated dishes to yield CDCs. Scale bar, 100 µm. (B) CDCs were previously characterized to be $CD105^+$ and $CD45^-$ (Cambier et al., 2017), the identity of CDCs was verified through quantifying the percentage of $CD105^+$ cells (> 90%) and $CD45^+$ cells (< 5%) (B), and evaluating the ability of CDCs to form secondary cardiospheres (cardiospheres^{2nd}) (C). For B, data are shown as mean ± SD from 4 biological replicates. For C, representative image from 3 biological replicates is shown.

Figure S2

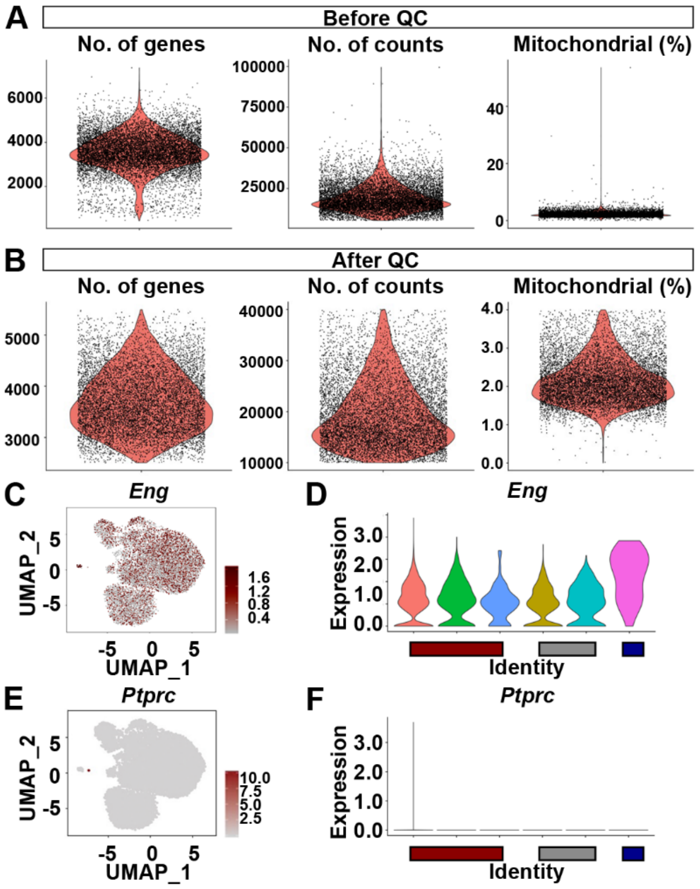

Figure S2. Quality control for scRNA-seq data. Violin plots illustrating the number of genes and counts, as well as the percent mitochondrial reads for cells before (A) and after (B) quality control. QC, quality control; No., number. Feature plot (C) and violin plot (D) showing the expression of *Eng* (encoding CD105) across 6 clusters in CDCs. Feature plot (E) and violin plot (F) showing the expression of *Ptprc* (encoding CD45) across 6 clusters in CDCs, confirming the CDCs harvested for scRNA-seq are mostly $CD105^{+}/CD45^{-}$ as described before (Cambier et al., 2017).

Figure S3

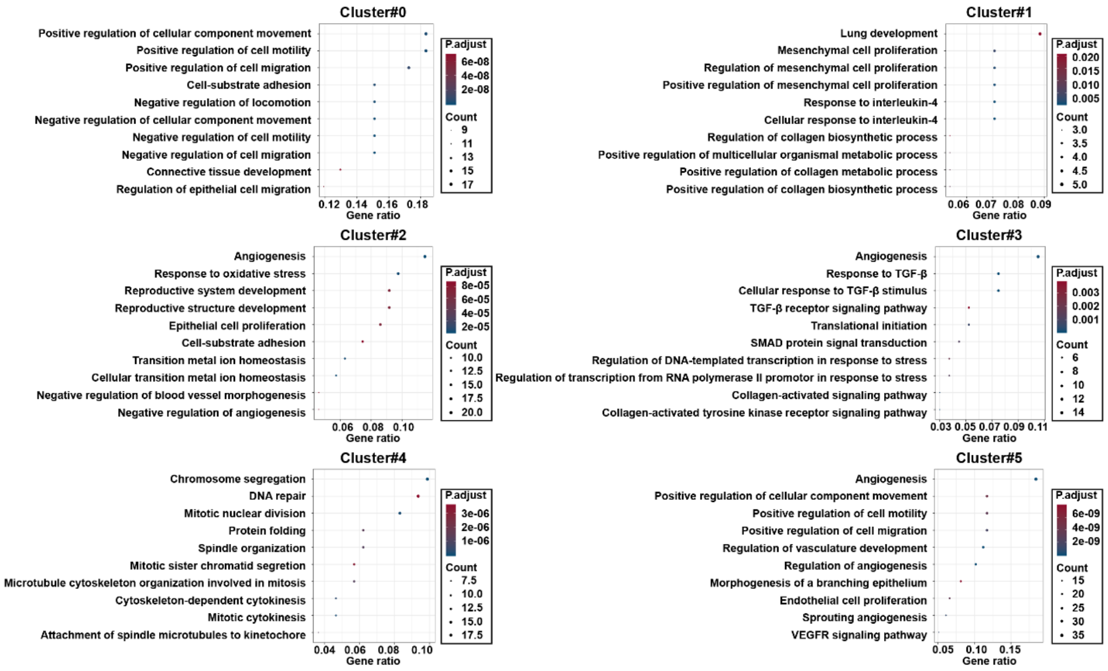

Fig. S3. Top 10 enriched GO categories of significantly upregulated differentially expressed genes for each cluster.

Figure S4

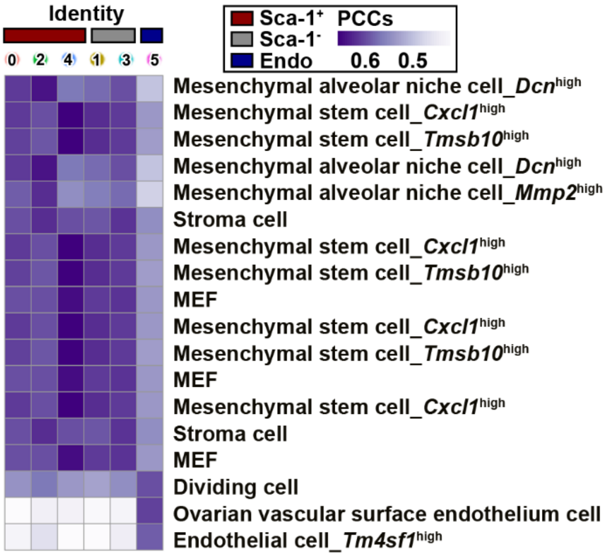

Figure S4. Heatmap showing the top 3 cellular identities for each cluster of CDCs via consulting with single-cell mouse cells atlas as quantified by Pearson correlation coefficients (PCCs). Correlation between subpopulations of CDCs and known mouse cell types were calculated (Sun et al., 2019), and cluster#5 was identified as endothelial cells, while other subpopulations of CDCs all correlate to various mesenchymal, stromal, and fibroblast cell types.

Figure S5

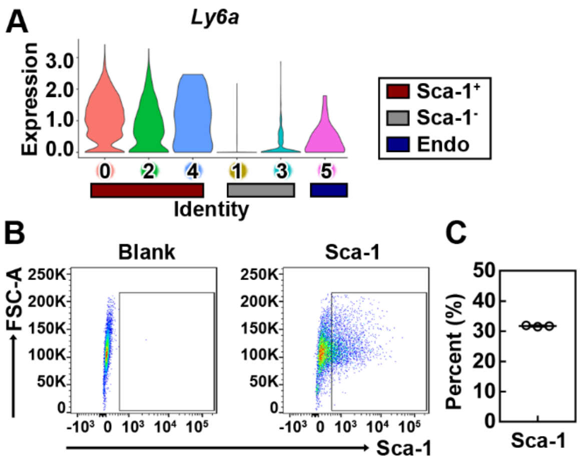

Figure S5. Quantification of Sca-1$^+$ cells within CDCs. (A) Violin plot showing the expression of *Ly6a* across 6 clusters in CDCs. Representative flow cytometry image (B) and quantification of the percentage of Sca-1$^+$ cells in CDCs (C). Data are shown as mean ± SD from 3 biological replicates.

Figure S6

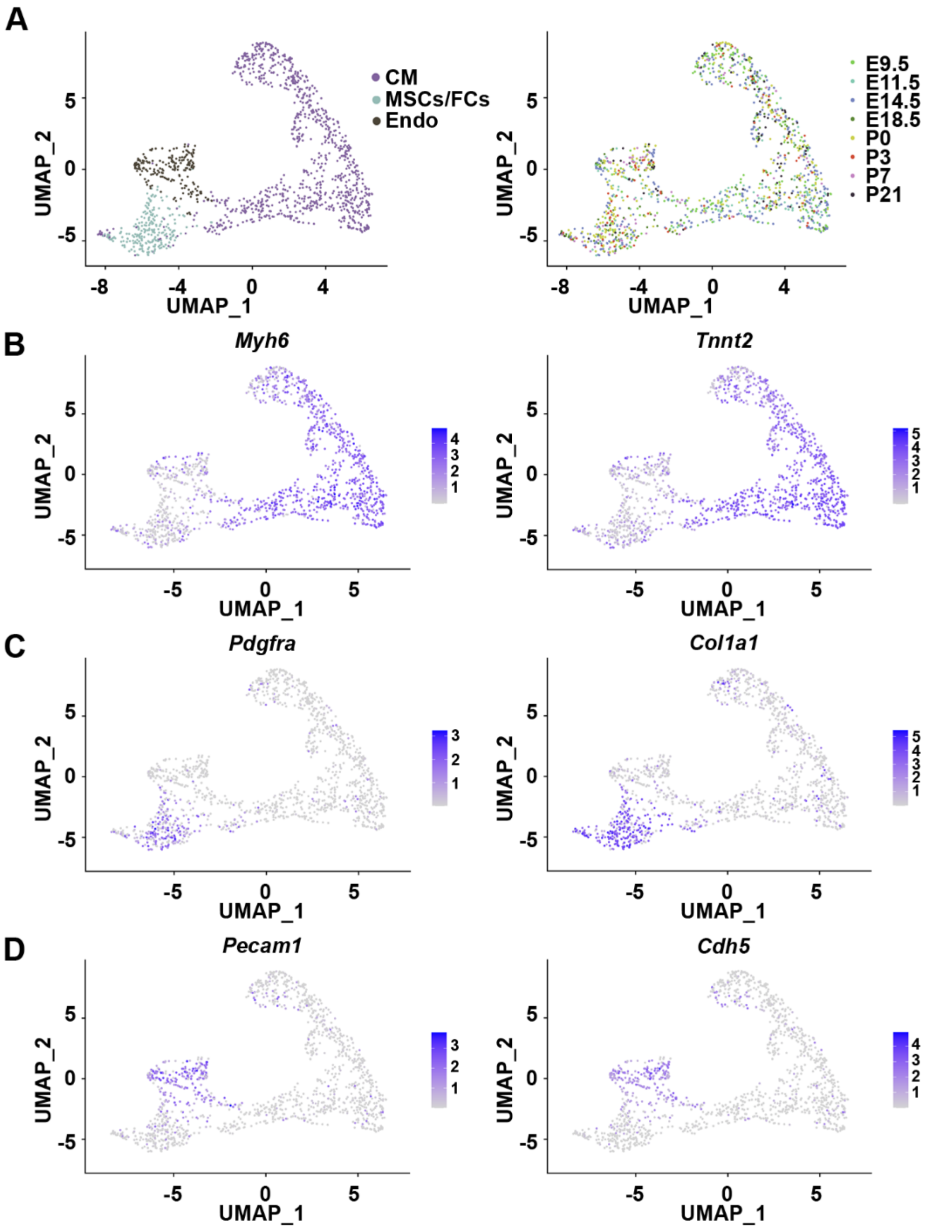

Figure S6. The atlas of three major cell types during mouse cardiac development. (A) UMAP plot revealed three major cardiac cell types including cardiomyocytes (CM), mesenchymal stromal cells/fibroblast-enriched cells (MSCs/FCs), and endothelial cells (Endo) during cardiac development. The corresponding cellular identity was verified via analyzing the distribution of marker genes of CM (*Myh6* and *Tnnt2*) (B), FCs (*Pdgfra* and *Col1a1*) (C), and Endo (*Pecam1* and *Cdh5*) (D).

Figure S7

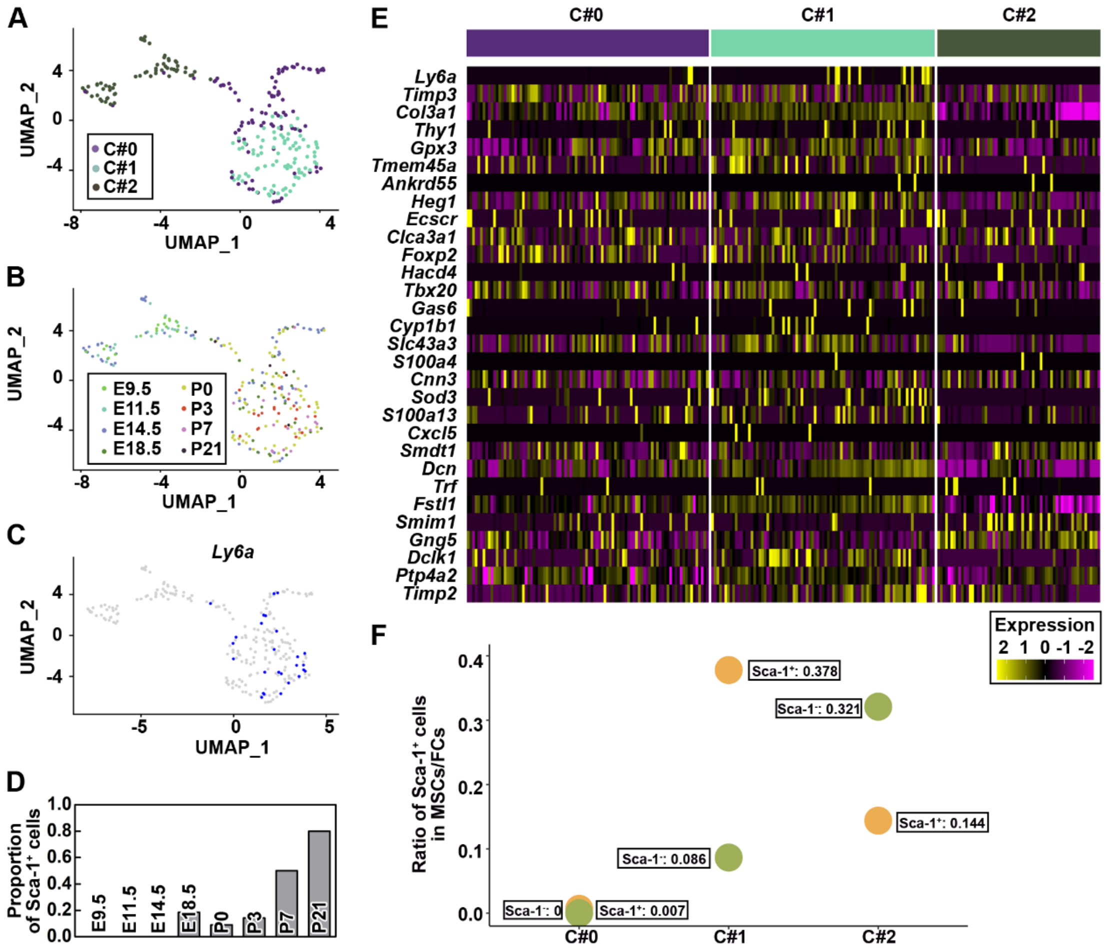

Figure S7. The atlas of three cellular subsets of MSCs/FCs during mouse cardiac development. (A-B) UMAP plot revealed three subsets of MSCs/FCs including cluster#0 (C#0), C#1, and C#2 during cardiac development. (C) Feature plot showing the distribution of *Ly6a*. (D) The proportion of Sca-1^+ cells among these subsets of MSCs/FCs was calculated during cardiac development. (E) Heatmap illustrating the similarity of gene signature of Sca-1^+ and Sca-1^- CDCs with three subsets of MSCs/FCs. (F) The ratio of Sca-1^+ cells in MSCs/FCs subsets was calculated.

Figure S8

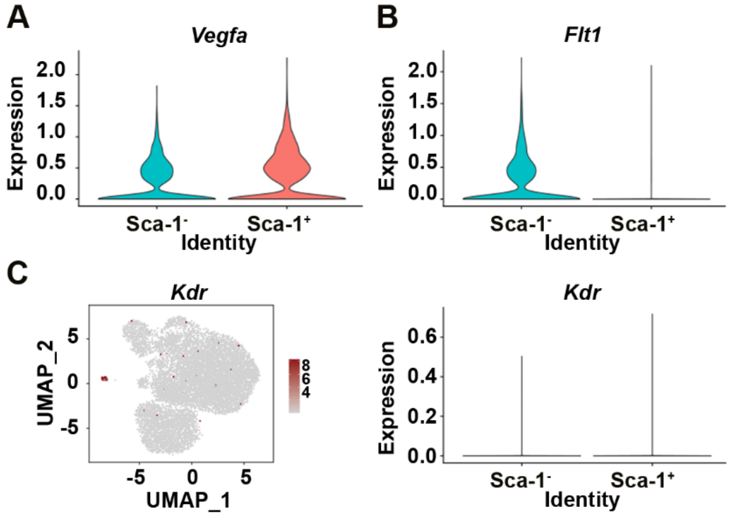

Figure S8. Expression analysis of *Vegfa* and the corresponding receptors. Violin plot showing the expression of *Vegfa* (A) and *Flt1* (B) in Sca-1$^-$ and Sca-1$^+$ CDCs based on scRNA-seq dataset. (C) The distribution and expression of *Kdr*, one of specific receptors of Vegfa, was shown by feature and violin plots, respectively.

Figure S9

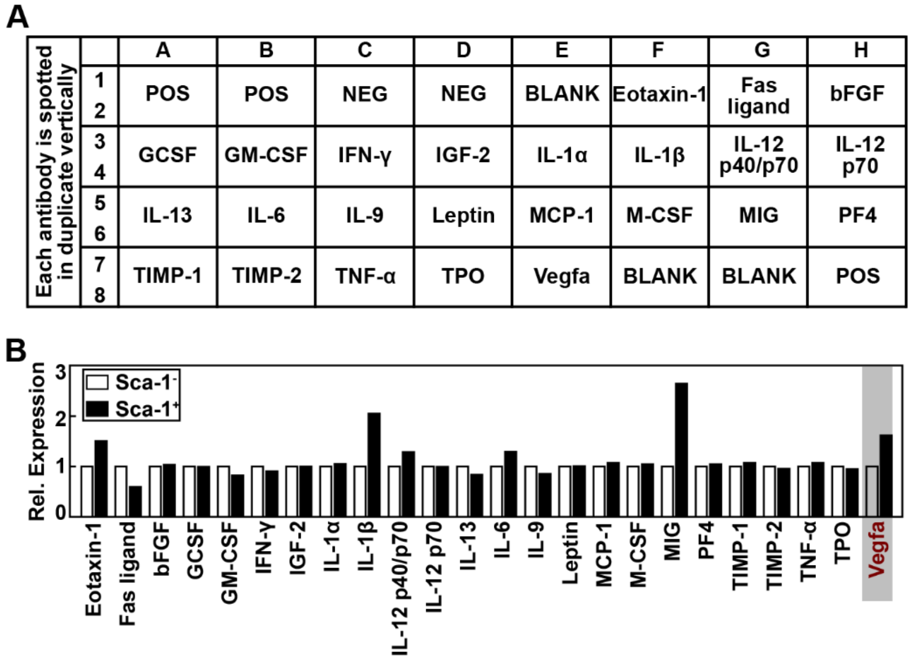

Figure S9. Angiogenesis-related protein array. (A) Arrangement of tested antibodies in the angiogenic array. (B) Relative (Rel.) expression of detected proteins from Sca-1$^-$ and Sca-1$^+$ CDCs, respectively.

Figure S10

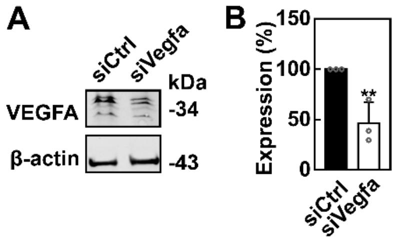

Figure S10. Expression analysis of Vegfa. Western blot analysis (A) and quantification (B) of Vegfa in Sca-1^+ CDCs after transfection with siVegfa for 3 days. Data are shown as mean ± SD from 3 independent experiments. *, significantly different from siCtrl; **, $p < 0.01$.

Figure S11

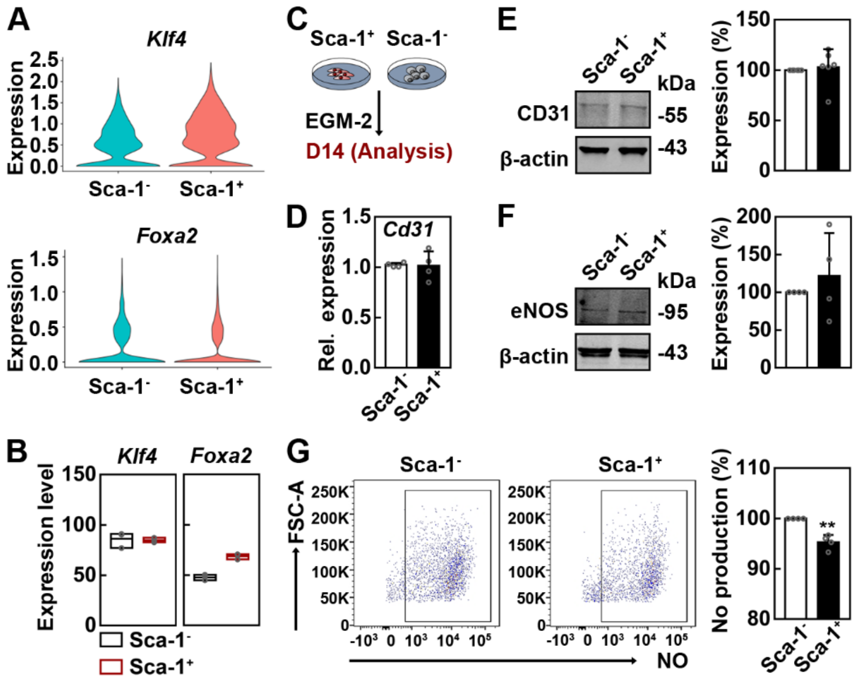

Figure S11. Evaluation of the endothelial differentiation capability of Sca-1⁻ and Sca-1⁺ CDCs. (A) Violin plot showing the expression of *Klf4* and *Foxa2* in Sca-1⁻ and Sca-1⁺ CDCs based on scRNA-seq dataset. (B) The expression of *Klf4* and *Foxa2* in Sca-1⁻ and Sca-1⁺ CDCs based on RNA-seq dataset. (C) Endothelial induction strategy by EGM-2 medium. After induction for 14 days, the expression of CD31 was assessed by qRT-PCR (D) and western blot (E); the expression of eNOS was assessed by western blot (F); the intracellular nitric oxide (NO) level was quantified by flow cytometry (G). For D, data are shown as mean ± SD from 4 biological replicates. For E, data are shown as mean ± SD from 6 independent experiments. For F-G, data are shown as mean ± SD from 4 independent experiments. *, significantly different from Sca-1⁻ CDCs; **, $p < 0.01$.

Figure S12

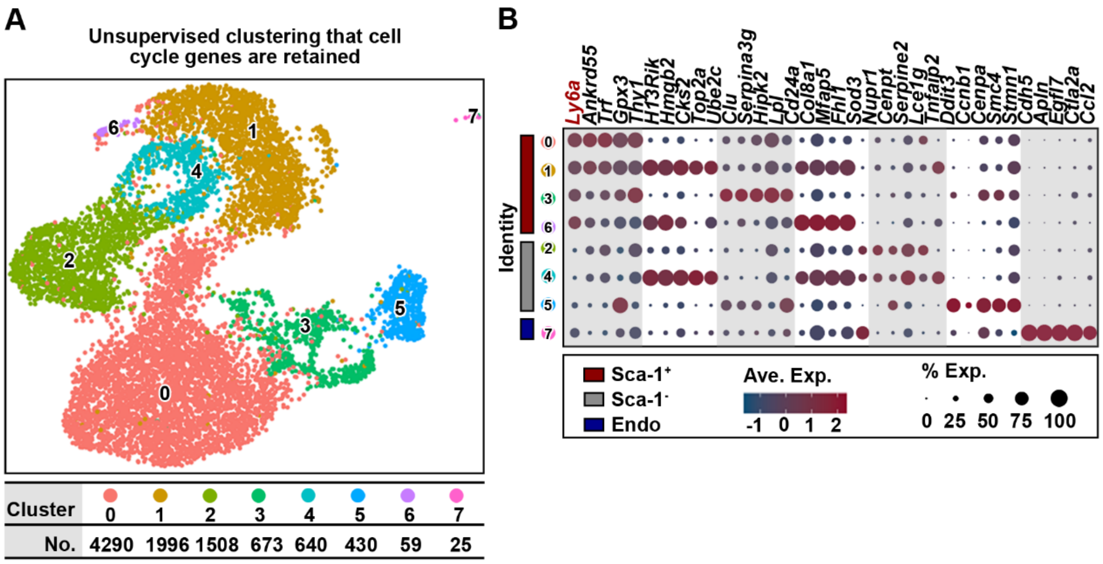

Figure S12. The cellular atlas of CDCs with cell cycle genes retained. (A) Unsupervised clustering revealed that CDCs contained 8 distinct clusters, in which cell cycle genes were retained. (B) Dot plot of marker genes for each cluster.

Figure S13

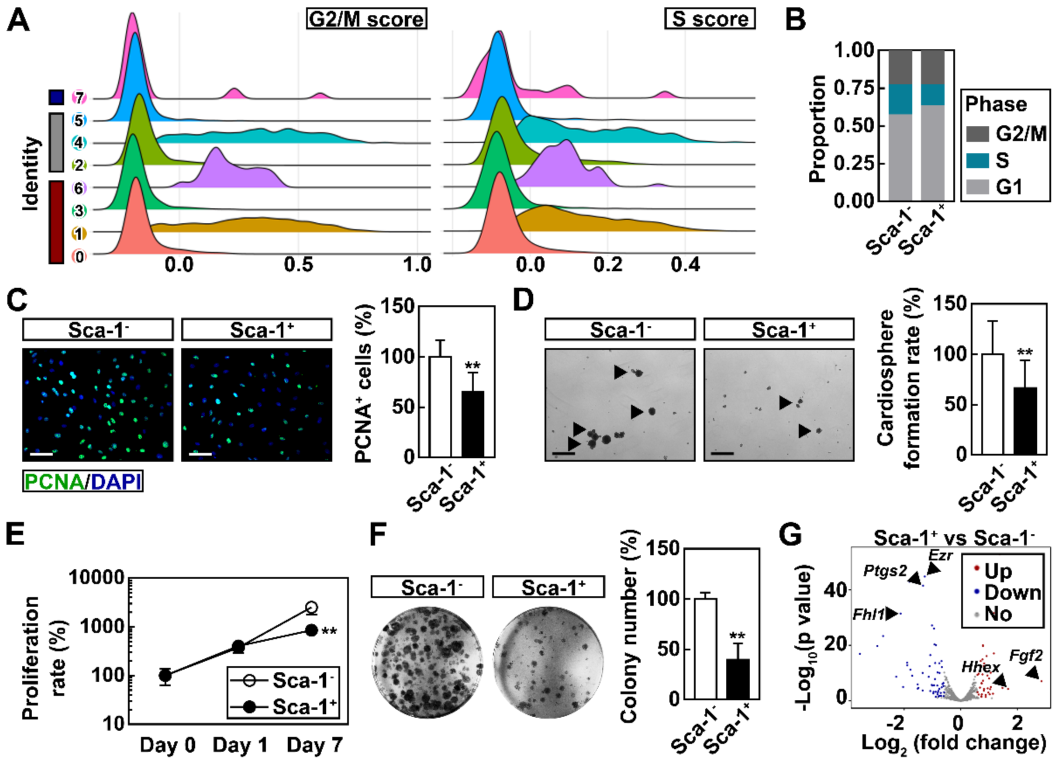

Figure S13. Evaluation of the proliferation capability of Sca-1⁻ and Sca-1⁺ CDCs. (A) Ridge plot showing the score distribution of G2/M and S phases for each subpopulations based on scRNA-seq dataset. (B) Quantification of the proportion of Sca-1⁻ and Sca-1⁺ CDCs at each cell cycle phase based on scRNA-seq dataset. (C) Representative immunofluorescence images of Sca-1⁻ and Sca-1⁺ CDCs from 2 biological replicates are shown. Cells were stained with PCNA (green); nuclei were labelled with DAPI (blue). Scale bar, 100 μm. Data are shown as mean ± SD. *, significantly different from Sca-1⁻ CDCs; **, $p < 0.01$. (D) Representative cardiosphere formation images of Sca-1⁻ and Sca-1⁺ CDCs from 3 biological replicates are shown. Scale bar, 100 μm. Data are shown as mean ± SD. *, significantly different from Sca-1⁻ CDCs; **, $p < 0.01$. (E) The proliferation rate of Sca-1⁻ and Sca-1⁺ CDCs on Day 0, Day 1, and Day 7 after being seeded as measured by CCK-8 assay. Data are shown as mean

± SD from 2 biological replicates. *, significantly different from Sca-1^{-} CDCs; **, $p < 0.01$. (F) Representative colony formation images of Sca-1^{-} and Sca-1^{+} CDCs from 4 biological replicates are shown. Scale bar, 100 μm. Data are shown as mean ± SD. *, significantly different from Sca-1^{-} CDCs; **, $p < 0.01$. (G) Volcano plot illustrating cell cycle phases-specific differentially expressed genes between Sca-1^{-} and Sca-1^{+} CDCs based on RNA-seq dataset. Red, significantly upregulated DEGs in Sca-1^{+} CDCs; blue, significantly downregulated DEGs in Sca-1^{+} CDCs; gray, no significant difference. Fold change ≥ 2 and $p < 0.05$ were considered significant.

Figure S14

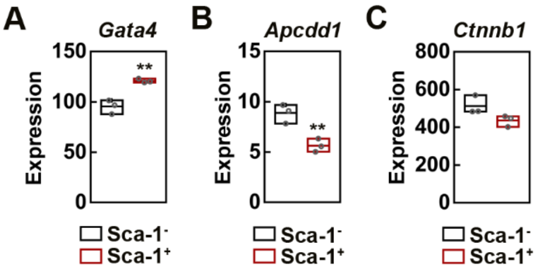

Figure S14. Expression analysis of Wnt signaling markers. The expression of *Gata4* (A), *Apcdd1* (B), and *Ctnnb1* (encoding β-catenin) (C), which were shown to be associated with CDCs' cardio-protective potency (Ibrahim et al., 2019), in Sca-1$^-$ and Sca-1$^+$ CDCs were analyzed based on RNA-seq dataset. Data are shown as mean ± SD from 3 biological replicates. *, significantly different from Sca-1$^-$ CDCs; **, $p < 0.01$.

Figure S15

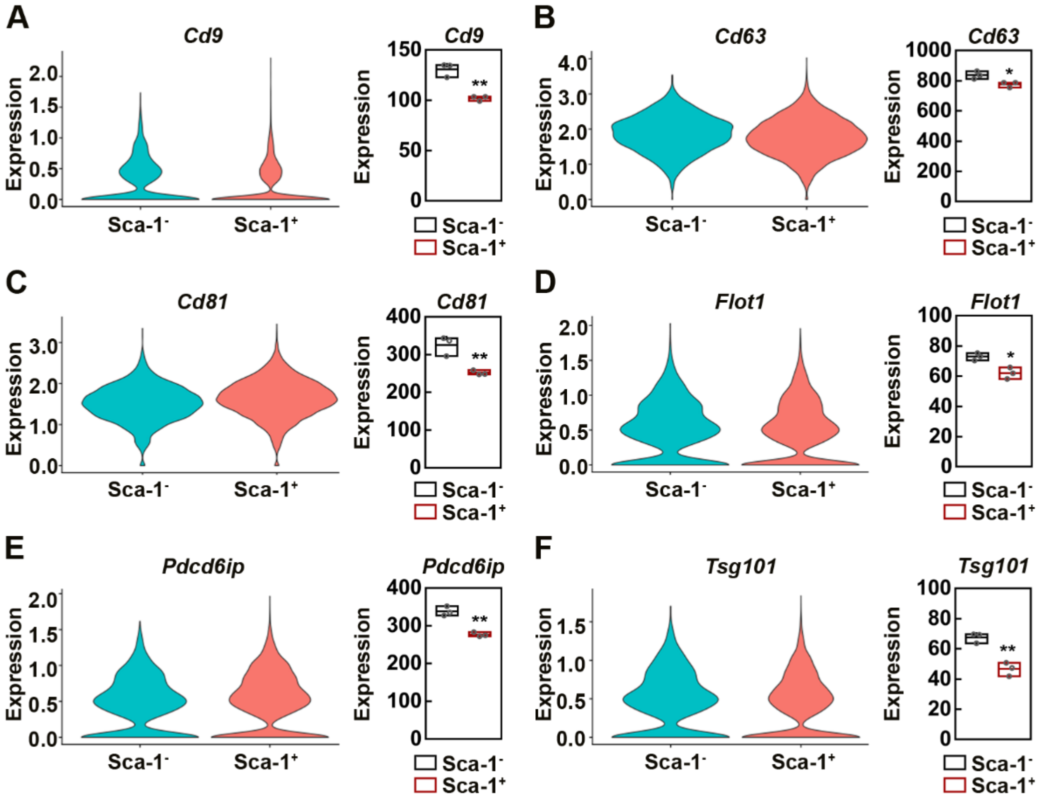

Figure S15. Expression analysis of exosomal markers. The expression exosomal markers (Kalluri and LeBleu, 2020) including *Cd9* (A), *Cd63* (B), *Cd81* (C), *Flot1* (D), *Pdcd6ip* (E), and *Tsg101* (F) in Sca-1$^-$ and Sca-1$^+$ CDCs based on scRNA-seq and RNA-seq datasets, respectively. For RNA-seq results, data are shown as mean ± SD from 3 biological replicates. *, significantly different from Sca-1$^-$ CDCs; *, $p < 0.05$; **, $p < 0.01$.

Table S1. Primers for qRT-PCR.

Gene	Forward	Reverse
Cd31	AGTTGCTGCCCATTCATCAC	CTGGTGCTCTATGCAAGCCT
Gapdh	AGGTCGGTGTGAACGGATTTG	GGGGTCGTTGATGGCAACA
